# Supplementary material for: Fluorescence lifetime Hong-Ou-Mandel sensing
Source: Nat Commun. 2023 Dec 4;14:8005. doi: 10.1038/s41467-023-43868-x (PMC10696080; doi:10.1038/s41467-023-43868-x)
Supplement: Supplementary file 1 — Supplementary Information [file 41467_2023_43868_MOESM1_ESM.pdf]

Supplementary Information: Fluorescence Lifetime Hong-Ou-Mandel Sensing

Ashley Lyons,[1] Vytautas Zickus,[1,2] Raúl Álvarez-Mendoza,[1] Danilo Triggiani,[3] Vincenzo Tamma,[3,4] Niclas Westerberg,[1] Manlio Tassieri,[5] and Daniele Faccio[1]

[1]*School of Physics and Astronomy, University of Glasgow, Glasgow, G12 8QQ, UK*
[2]*Department of Laser Technologies, Center for Physical Sciences and Technology, LT-10257, Vilnius, Lithuania*
[3]*School of Mathematics and Physics, University of Portsmouth, Portsmouth, PO1 3QL, UK*
[4]*Institute of Cosmology and Gravitation, University of Portsmouth, Portsmouth PO1 3FX, UK*
[5]*James Watt School of Engineering, University of Glasgow, Glasgow, G12 8QQ, UK*

I. ANALYTICAL MODEL OF THE FL-HOM APPROACH

Inspired by Refs. [20, 21] of the main text, the Gaussian reference signal $E_{\rm ref}(t)$ and the signal $E_{\rm FP}(t)$ of the fluorescent pulse can be written respectively as

$$E_{\rm ref}(t) = G_\sigma(t)\mathrm{e}^{-\mathrm{i}\omega_0 t} \equiv G_0 \mathrm{e}^{-\frac{t^2}{2\sigma^2}}\mathrm{e}^{-\mathrm{i}\omega_0 t}, \tag{1a}$$

$$E_{\rm FP}(t) = S_\mu(t)\mathrm{e}^{-\mathrm{i}\omega_0 t}\mathrm{e}^{\mathrm{i}\Phi(t)} \equiv S_0 \mathrm{e}^{-\frac{t}{2\mu}}\theta(t)\mathrm{e}^{-\mathrm{i}\omega_0 t}\mathrm{e}^{\mathrm{i}\Phi(t)}, \tag{1b}$$

where G_0 and S_0 are the magnitudes and ω_0 the frequency of the signal and of the reference (both supposed monochromatic), and $\theta(t)$ is the Heaviside step function. Here $\Phi(t)$ represents a random relative phase with a Lorentzian distribution such that $\left\langle \mathrm{e}^{-\mathrm{i}\Phi(t+\tau)}\mathrm{e}^{\mathrm{i}\Phi(t)}\right\rangle = \exp(-|\tau|/t_c)$, whose coherence time t_c we will assume is of the order of μ (the fluorescence lifetime), and $\left\langle \mathrm{e}^{\mathrm{i}\Phi(t+\tau)}\mathrm{e}^{\mathrm{i}\Phi(t)}\right\rangle = 0$. In Eq. (1) we also assumed that the excitation pulse is much shorter than the lifetime μ of the fluorescence, so that it is well approximated with a delta function in the convolution operation in Eq.(1) in the main text. As such, we want to emphasise that what follows is only valid when $\sigma_{\rm exc} \ll \mu$ (where $\sigma_{\rm exc}$ is the excitation pulse duration), as is the case in the experiment.

Once we introduce a relative delay τ between the interferometer arms, the fields $E_i(t)$, with $i = 1, 2$, at the i-th detector, will be given by

$$\begin{pmatrix} E_1(t) \\ E_2(t) \end{pmatrix} = \begin{pmatrix} \sqrt{T} & \mathrm{i}\sqrt{R} \\ \mathrm{i}\sqrt{R} & \sqrt{T} \end{pmatrix} \begin{pmatrix} E_{\rm ref}(t-\tau) \\ E_{\rm FP}(t) \end{pmatrix} = \begin{pmatrix} \sqrt{T}E_{\rm ref}(t-\tau) + \mathrm{i}\sqrt{R}E_{\rm FP}(t) \\ \mathrm{i}\sqrt{R}E_{\rm ref}(t-\tau) + \sqrt{T}E_{\rm FP}(t) \end{pmatrix} \tag{2}$$

where R and T are the reflectance and transmittance of the final beamsplitter of the interferometer respectively. We can thus evaluate from Eqs. (1)-(2) the second-order normalised correlation function associated with detections at times t_1 and $t_2 = t_1 + t'$ at the two detectors

$$g^{(2)}(t_1, t'; \tau) = \frac{\langle E_1(t_1)E_2(t_1+t')E_2^*(t_1+t')E_1^*(t_1)\rangle}{\langle E_1(t_1)E_1^*(t_1)\rangle\langle E_2(t_1+t')E_2^*(t_1+t')\rangle} = \frac{\langle I_1(t_1)I_2(t_1+t')\rangle}{\langle I_1(t_1)\rangle\langle I_2(t_1+t')\rangle}, \tag{3}$$

where $\langle\cdot\rangle$ denotes the average over the statistical ensemble consisting of multiple fluorescence events, and $I_i(t_i) = E_i(t_i)E_i^*(t_i) \equiv |E_i(t_i)|^2$. We easily obtain

$$I_1(t_1) = TG_\sigma(t_1-\tau)^2 + RS_\mu(t_1)^2 - \mathrm{i}\sqrt{RT}G_\sigma(t_1-\tau)S_\mu(t_1)\mathrm{e}^{-\mathrm{i}\omega_0\tau}\mathrm{e}^{-\mathrm{i}\Phi(t_1)} + \mathrm{i}\sqrt{RT}G_\sigma(t_1-\tau)S_\mu(t_1)\mathrm{e}^{\mathrm{i}\omega_0\tau}\mathrm{e}^{\mathrm{i}\Phi(t_1)}, \tag{4a}$$

$$I_2(t_2) = RG_\sigma(t_2-\tau)^2 + TS_\mu(t_2)^2 + \mathrm{i}\sqrt{RT}G_\sigma(t_2-\tau)S_\mu(t_2)\mathrm{e}^{-\mathrm{i}\omega_0\tau}\mathrm{e}^{-\mathrm{i}\Phi(t_2)} - \mathrm{i}\sqrt{RT}G_\sigma(t_2-\tau)S_\mu(t_2)\mathrm{e}^{\mathrm{i}\omega_0\tau}\mathrm{e}^{\mathrm{i}\Phi(t_2)}, \tag{4b}$$

and thus, since $\langle \mathrm{e}^{\mathrm{i}\Phi(t)}\rangle = 0$, averaging yields

$$\langle I_1(t_1)\rangle = TG_\sigma(t_1-\tau)^2 + RS_\mu(t_1)^2, \tag{5a}$$

$$\langle I_2(t_1+t')\rangle = RG_\sigma(t_1+t'-\tau)^2 + TS_\mu(t_1+t')^2. \tag{5b}$$

The numerator of the second-order correlation function in Eq. (3) can be separated as the sum of two contributions, where we have neglected terms containing only one instance of the random phase (since $\langle \mathrm{e}^{\mathrm{i}\Phi(t)}\rangle = 0$):

$$\langle I_1(t_1)I_2(t_1+t')\rangle = B(t_1, t') + K(t_1, t'), \tag{6}$$

with

$$B(t_1,t') = (TG_\sigma(t_1-\tau)^2 + RS_\mu(t_1)^2)(RG_\sigma(t_1+t'-\tau)^2 + TS_\mu(t_1+t')^2) \equiv \langle I_1(t_1)\rangle\langle I_2(t_1+t')\rangle, \tag{7a}$$

$$K(t_1,t') = \Big\langle \Big(-\mathrm{i}\sqrt{RT}G_\sigma(t_1-\tau)S_\mu(t_1)\mathrm{e}^{-\mathrm{i}\omega_0\tau}\mathrm{e}^{-\mathrm{i}\Phi(t_1)} + \mathrm{i}\sqrt{RT}G_\sigma(t_1-\tau)S_\mu(t_1)\mathrm{e}^{\mathrm{i}\omega_0\tau}\mathrm{e}^{\mathrm{i}\Phi(t_1)}\Big) \times \tag{7b}$$
$$\times \Big(+\mathrm{i}\sqrt{RT}G_\sigma(t_1+t'-\tau)S_\mu(t_1+t')\mathrm{e}^{-\mathrm{i}\omega_0\tau}\mathrm{e}^{-\mathrm{i}\Phi(t_1+t')} - \mathrm{i}\sqrt{RT}G_\sigma(t_1+t'-\tau)S_\mu(t_1+t')\mathrm{e}^{\mathrm{i}\omega_0\tau}\mathrm{e}^{\mathrm{i}\Phi(t_1+t')}\Big)\Big\rangle.$$

Once again, we can simplify Eq. (7b) by recalling that $\langle \mathrm{e}^{\pm\mathrm{i}\Phi(t_1+t')}\mathrm{e}^{\pm\mathrm{i}\Phi(t_1)}\rangle = 0$ and $\langle \mathrm{e}^{\pm\mathrm{i}\Phi(t_1)}\mathrm{e}^{\pm\mathrm{i}\Phi(t_1)}\rangle = 0$, whereas $\langle \mathrm{e}^{\mp\mathrm{i}\Phi(t_1+t')}\mathrm{e}^{\pm\mathrm{i}\Phi(t_1)}\rangle = \exp(-|t'|/t_c)$. We thus obtain

$$\begin{aligned} K(t_1,t') &= -RTG_\sigma(t_1-\tau)G_\sigma(t_1+t'-\tau)S_\mu(t_1)S_\mu(t_1+t')\left\langle \mathrm{e}^{-\mathrm{i}\Phi(t_1)}\mathrm{e}^{\mathrm{i}\Phi(t_1+t')}\right\rangle + \text{c.c.} \\ &= -2RTG_\sigma(t_1-\tau)G_\sigma(t_1+t'-\tau)S_\mu(t_1)S_\mu(t_1+t')e^{-|t'|/t_c}, \end{aligned} \tag{8}$$

Substituting Eqs. (5)-(8) into Eq. (3), we finally obtain

$$g^{(2)}(t_1,t';\tau) = 1 - 2\frac{RTG_\sigma(t_1-\tau)G_\sigma(t_1+t'-\tau)S_\mu(t_1)S_\mu(t_1+t')e^{-|t'|/t_c}}{(TG_\sigma(t_1-\tau)^2 + RS_\mu(t_1)^2)(RG_\sigma(t_1+t'-\tau)^2 + TS_\mu(t_1+t')^2)} \tag{9}$$

Whilst infinitely temporally precise detectors would measure $g^{(2)}$ as seen in Eq. (9), realistic detectors acquire for some time $T_{\text{acquisition}}$ and have some finite resolution time within which a coincidence is measured $T_{\text{resolution}}$. As such, the coincidence signal takes the form

$$C_{\text{signal}}(\tau) = \int_{-T_{\text{acquisition}}/2}^{T_{\text{acquisition}}/2} dt_1 \int_{-T_{\text{resolution}}/2}^{T_{\text{resolution}}/2} dt' \,\langle I_1(t_1)I_2(t_1+t')\rangle\,. \tag{10}$$

Typically, and as is the case here, both $T_{\text{acquisition}}$ and $T_{\text{resolution}}$ are larger than any other physical timescale, and we can therefore proceed in the calculations by setting these to infinity. Furthermore, for simplicity, we will normalise this with respect to $\tau \to \infty$ such that $C(\tau) = C_{\text{signal}}(\tau)/C_{\text{signal}}(\infty)$, to reflect what is typically done in experiments. This yields

$$C(\tau) = 1 - e^{-\tau/\mu}\left(\frac{4\pi RTG_0^2S_0^2\sigma^2\exp\left[\sigma^2\left(\frac{1}{4\mu^2}+\frac{1}{t_c^2}\right)\right]\left[\operatorname{erf}(\tau/\sigma+\sigma/t_c-\sigma/2\mu)-\operatorname{erf}(\sigma/t_c)\right]}{RT\left(\pi\sigma^2G_0^4+\mu^2S_0^4\right)+e^{-\sigma^2/4\mu^2}\sqrt{\pi}\mu\sigma G_0^2S_0^2\left(R^2+T^2\right)}\right)\theta(\tau-\sigma^2/2\mu), \tag{11}$$

$$= 1 - 4C_0(\sigma)e^{-\tau/\mu}, \tag{12}$$

the last line of which coincides with Eq. (3) in the main text and uses the assumption that $\sigma \ll \mu$. Within the calculation we have made the approximation of $\operatorname{erf}(x/\sigma) \simeq \operatorname{sign}(x/\sigma)$ for small σ. This introduces some error, negligible when $\tau \gg \sigma$ which is the regime in which we are interested. Note that Eq. (11) is still within a few percent accurate when $\tau \sim 2\sigma$, as confirmed by numeric integration. This is in line with the approximation of the same kind at the start of this calculation. Finally, we can expand to first order in σ (with respect to the other timescales where we recall that $t_c \sim \mu$) when $\tau > 0$, yielding the simplified form

$$C(\tau) \sim 1 - e^{-\tau/\mu}\left(\frac{4\sigma^2\pi G_0^2}{\mu^2S_0^2}\right) = 1 - 4C_0^{\text{approx}}e^{-\tau/\mu}. \tag{13}$$

To ensure that the visibility is accurate in Fig. 4 (b) of the main text, we instead numerically integrate part of the calculation, circumventing the approximation of $\operatorname{erf}(x/\sigma) \simeq \operatorname{sign}(x/\sigma)$. This ensures that the visibility is accurately calculated also when $\sigma \gg \mu$.

II. STATISTICAL UNCERTAINTY FOR COINCIDENT MEASUREMENTS

Here, we evaluate how the statistical fluctuations of the photon count measurements affects the uncertainty of the measured number of coincident events. First, we begin with the assumption that the photon arrival statistics at each of the two detectors are Poissonian distributed and act independently i.e. the rate of correlated events between the

two detectors can also be modelled using Poissonian statistics (in contrast to quantum light sources such as using SPDC). In this case the average number of coincident events per second, C, will be given by:

$$C = \frac{I_1 I_2}{R.R.}, \tag{14}$$

where $I_{1,2}$ are the mean count rates for detector 1 and 2, and $R.R.$ is the repetition rate of the laser. The variance of coincidence counts, $\mathrm{Var}(C)$, can then be calculated by taking [1]

$$\mathrm{Var}(C) = \mathrm{Var}(I_1 I_2) = \left(\sigma_1^2 + I_1^2\right)\left(\sigma_2^2 + I_2^2\right) - I_1^2 I_2^2, \tag{15}$$

where $\sigma_{1,2}$ are the standard deviations of the count rates at the two detectors. Assuming that both detectors register approximately the same number of photon counts N and we set the standard deviation to be $\sqrt{N}$ following Poissonian statistics, it follows that

$$\begin{aligned}\mathrm{Var}(C) &= \left(N + N^2\right)\left(N + N^2\right) - N^2 N^2 \\ &= N^4 + 2N^3 + N^2 - N^4 \\ &= N^2 + 2N^3. \end{aligned} \tag{16}$$

From this we can take the standard deviation of the coincidences

$$\sigma_C = N\sqrt{1 + 2N} \tag{17}$$

and the Signal to Noise Ratio (SNR), which we define as the mean divided by the standard deviation

$$\mathrm{SNR}_C = \frac{N}{\sqrt{1 + 2N}}. \tag{18}$$

This then informs how the noise in the coincidence counts scales as the measurement time is increased, equivalent to increasing N. For very small N, the SNR will increase approximately linearly but will tend towards the $\sqrt{N}$ scaling for larger N. We verify this by tuning the optical delay of our interferometer to a region where we do not expect to observe interference, take many example measurements of short exposure time which can then be summed together to model longer exposure times (see below). We then fit Eq. 18 with an added proportionality term to account for any potential additional measurement factors, Fig. 1. We note that for $N > 1$, $\mathrm{SNR}_C \sim \sqrt{N}/\sqrt{2}$, i.e. the SNR scales as a standard intensity measurement albeit with a reduction of $\sqrt{2}$.

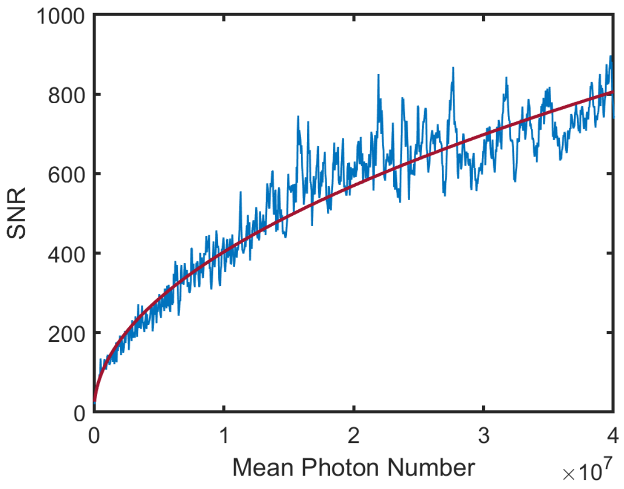

FIG. 1. SNR of the coincidence counts as a function of the average number of photons detected or, equivalently, the measurement duration. Blue shows experimental data whilst red shows a fit to Eq. 18.

III. EFFECTS OF SAMPLE TEMPERATURE ON THE LIFETIME

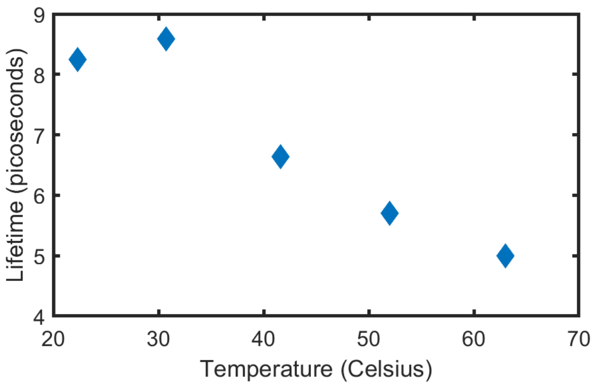

FIG. 2. Lifetime variation as a function of temperature for the 4-DASPI sample in water. Error bars not visible due to relatively small size.

Here, we show our approach's potential as a temperature probing technique using the fluorescence lifetime. The 4-DASPI sample is used and the cuvette holding the solution is heated from room temperature to $63°C$ using a resistive heating strip. Lifetimes in a range of approximately 8.25 - 5 ps were observed, shown in Figure 2.

IV. SAMPLE DATA WITH DIFFERENT ACQUISITION TIMES

Below, in Figure 3, we show example normalised $g^{(2)}$ curves obtained for different acquisition times along with the fits to our model to obtain the lifetime as described in the main paper. Data was obtained in short acquisitions of 50 ms for each optical delay with and summed together to produce longer exposures. For longer acquisition times the short exposure measurements were added together in different combinations to produce more statistical samples. The optical delay sampling (range and number of steps) for each acquisition time was kept constant.

V. AVERAGE PARTICLE INTERACTION

We note that the concentration of fluorescent dyes used in this study (or order mM) are relatively high and it is thus important to discern whether or not interaction between fluorescent molecules occur which can potentially affect the lifetime. Firstly, we estimate the average distance between fluorescent molecules as

$$x = \frac{0.55}{n^{-1/3}} \tag{19}$$

following [2] where n is the concentration in particles per unit volume. For our highest concentration sample at $\sim$ 5 mM, this yields $x \approx 3.8$ nm. Next, we evaluate the average distance a fluorescent molecule moves whilst in the excited state, which we define using the lifetime. Assuming Brownian motion, this can be calculated as

$$\delta x = \sqrt{2D\mu} \tag{20}$$

with D as the translational diffusion coefficient.

$$D = \frac{k_B T}{6\pi\eta r} \tag{21}$$

where k_B is the Boltzmann constant, T the absolute temperature, η the viscosity, and r the average particle radius [2]. Taking $T = 293$ K, $\eta = 1$ mPa·s (corresponding to water), and $r = 0.5$ nm, we find that $D = 430$ nm$^2/\mu$s. This results in a δx of 0.1 nm. As this is significantly smaller than the average distance between particles, it is reasonable to conclude that there is no interaction between excited fluorophores and neighbouring molecules.

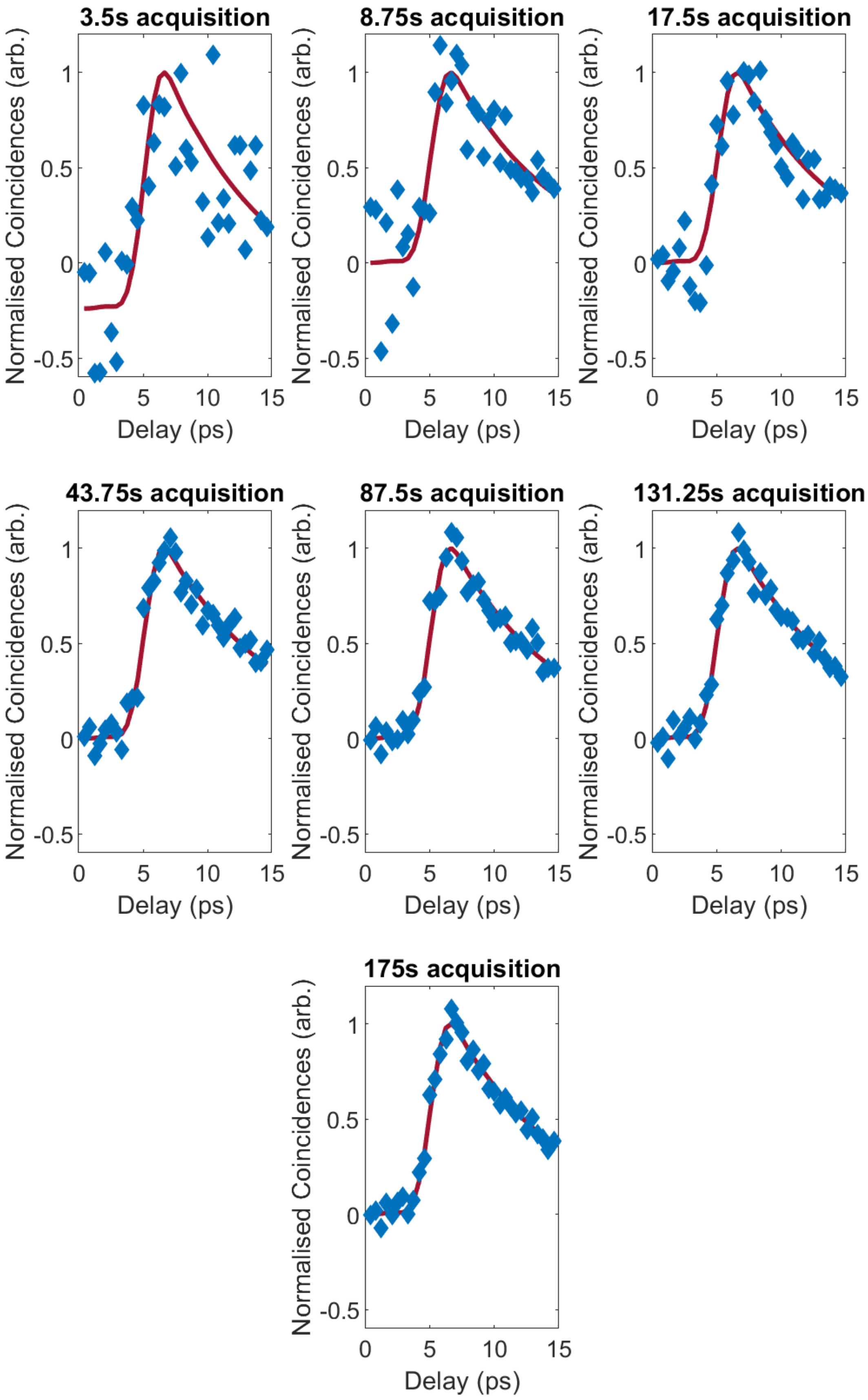

FIG. 3. Experimental data and associated fits for a range of acquisition times. Blue shows experimental data points and red shows the result of the MCMC-based fitting procedure discussed in the main paper.

VI. VISCOSITY MEASUREMENTS OF 4-DASPI ON LOG SCALE

Below we plot the same data as shown in Fig. 5 of the main paper on a log-log scale, often used as calibration for molecular rotor probes for viscosity. The red line shows a linear fit to this data, yielding a gradient of 1.377. Typical values found in literature for this gradient are around 0.67 which is in line with predictions from the Förster-Hofmann relation [3]. It is known however that this relation only holds for intermediate viscosities and not at the low viscosity range explored here [4, 5].

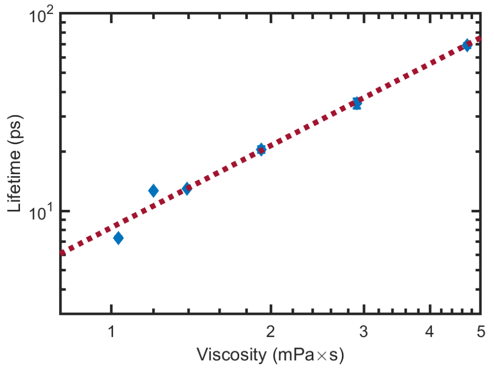

FIG. 4. Measured lifetime vs viscosity of the 4-DASPI probe on a log-log scale. Red shows a linear fit with a gradient of 1.377.

VII. LIFETIME MEASUREMENT OF 4-DASPI IN ETHANOL

In order to compare the FL-HOM approach against lifetime values found through other methods we measured the fluorescence signal of the 4-DASPI dye in ethanol. Fig.5 shows the normalised number of coincidence events as a function of the optical delay, τ, where the negative of the coincidences are plotted on a log scale in much the same manner as Fig.3 of the main paper. The red line shows a tail fit to a single exponential yielding a lifetime of 65.2 ± 1.2 ps. This is in close agreement with the work of Sibbett et al. [6].

[1] Leo A. Goodman. On the exact variance of products. *Journal of the American Statistical Association*, 55:708, 12 1960.

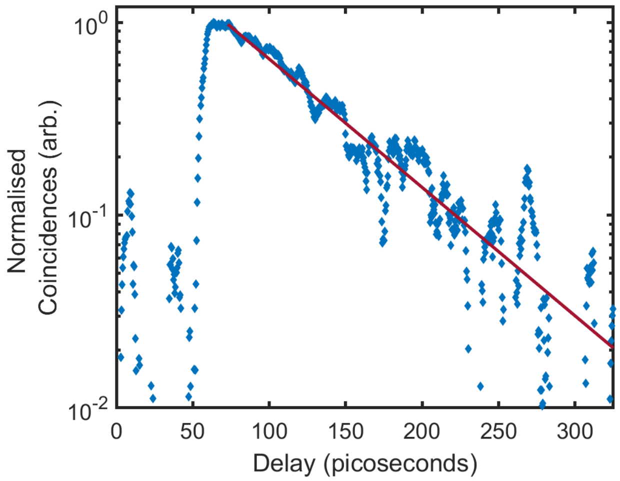

FIG. 5. Fluorescence signal of 4-DASPI in ethanol measured using the FL-HOM approach.

[2] S. Chandrasekhar. Stochastic problems in physics and astronomy. *Reviews of Modern Physics*, 15:1–89, 1 1943.

[3] Th. Förster and G. Hoffmann. Die viskositätsabhängigkeit der fluoreszenzquantenausbeuten einiger farbstoffsysteme. *Zeitschrift für Physikalische Chemie*, 75:63–76, 7 1971.

[4] James A. Levitt, Marina K. Kuimova, Gokhan Yahioglu, Pei-Hua Chung, Klaus Suhling, and David Phillips. Membrane-bound molecular rotors measure viscosity in live cells via fluorescence lifetime imaging. *The Journal of Physical Chemistry C*, 113:11634–11642, 7 2009.

[5] Marina K. Kuimova. Mapping viscosity in cells using molecular rotors. *Physical Chemistry Chemical Physics*, 14:12671, 10 2012.

[6] W. Sibbett and J.R. Taylor. Passive mode locking in the blue spectral region. *Optics Communications*, 46:32–36, 6 1983.
